# Role of Kir2.1 in human monocyte‐derived foam cell maturation

**DOI:** 10.1111/jcmm.12705

**Published:** 2015-12-22

**Authors:** Wei Zhang, Xin‐Jun Lei, Yi‐Fan Wang, Dong‐Qi Wang, Zu‐Yi Yuan

**Affiliations:** ^1^ Department of NeonatologyFirst Affiliated Hospital of Xi'an Jiaotong UniversityXi'anShaanxiChina; ^2^ Department of CardiologyFirst Affiliated Hospital of Xi'an Jiaotong UniversityXi'anShaanxiChina; ^3^ Key Laboratory of Environment and Gene Related to Diseases of Ministry of EducationXi'an Jiaotong UniversityXi'anShaanxiChina

**Keywords:** potassium channels, monocytes, macrophages, foam cells, atherosclerosis

## Abstract

The role of K^+^ channels in macrophage immunomodulation has been well‐established. However, it remains unclear whether K^+^ channels are involved in the lipid uptake of macrophages. The expression and function of the inward rectifier potassium channel (Kir2.1, KCNJ2) in Human acute monocytic leukemia cell line (THP‐1) cells and human monocytes derived macrophages (HMDMs) were investigated using RT‐PCR and western blotting, and patch clamp technique. The expression of scavenger receptors in THP‐1–derived macrophages was detected using western blotting. Expressions of Kir2.1 mRNA and protein in HMDMs were significantly decreased by 60% (*P* < 0.05) and 90% (*P* < 0.001) on macrophage maturation, but overexpressed by approximately 1.3 (*P* > 0.05) and 3.8 times (*P* = 0.001) after foam cell formation respectively. Concurrently, the Kir2.1 peak current density in HMDMs, mature macrophages and foam cells, measured at −150 mV, were −22.61 ± 2.1 pA/pF, −7.88 ± 0.60 pA/pF and −13.39 ± 0.80 pA/pF respectively (*P* < 0.05). In association with an up‐regulation of Kir2.1 in foam cells, the SR‐A protein level was significantly increased by over 1.5 times compared with macrophages (*P* < 0.05). THP‐1 cells contained much less lipids upon Kir2.1 knockdown and cholesterol ester/total cholesterol ratio was 29.46 ± 2.01% (*P* < 0.05), and the SR‐BI protein level was increased by over 6.2 times, compared to that of macrophages (*P* < 0.001). Kir2.1 may participate in macrophage maturation and differentiation, and play a key role in lipid uptake and foam cell formation through modulating the expression of scavenger receptors.

## Introduction

Atherosclerosis (AS) is a progressive, multifactorial, multisystemic, chronic, inflammatory disease characterized by aberrant vascular homeostasis, cytokine network dysfunction and cell biological behaviour disorders 1, 2. Endothelial cell activation and dysfunction are crucial initiating events in AS plaque formation 3, 4, which facilitate low‐density lipoprotein (LDL) into the tunica intima to be oxidized as oxidized LDL (ox‐LDL) 4, 5. ox‐LDL can further promote atherosclerotic plaque initiation and progression, induce macrophage‐to‐foam cell transformation, and facilitate the migration of smooth muscle cells into the tunica intima and their proliferation 5, 6, 7, which also facilitate endothelial cell dysfunction.

The formation of foam cells, a critical early event in the pathogenesis of AS, is associated with the disruption of normal macrophage cholesterol metabolism, involving cholesterol influx, esterification and efflux 8. Macrophage scavenger receptor class A (SR‐A) and CD36 are the main receptors for ox‐LDL uptake in association with foam cell formation 9. But scavenger receptor class B type I (SR‐BI) along with ATP‐binding cassette transporters A1(ABCA1) and G1 (ABCG1) play crucial roles in promoting cholesterol efflux from cells to lipid‐poor apolipoprotein A‐I 10. When the expression of molecule responsible for cholesterol influx and esterification increases 11, 12, 13 and/or molecule for its efflux decrease 14, macrophages uptake ox‐LDL unlimitedly and reduce cholesterol efflux, thus ultimately transforming into lipid‐laden foam cells.

Voltage‐dependent potassium channels are one of the important regulators during macrophage maturation, activation and differentiation 15, 16, 17, 18, 19. A previous study indicated that both the inward rectifier potassium (Kir) channel) Kir2.1 (KCNJ2) and the outward delayed rectifier potassium channel Kv1.3 are involved in murine bone marrow‐derived macrophages (BMDM) proliferation and differentiation 15. It has been demonstrated that a negative shift (hyperpolarization) in the resting potential is an initiation 20 of the differentiation for macrophages 21, 22. The balance between Kir2.1 and Kv1.3 in macrophages determines the resting potentials, and is thereby involved in proliferation and activation 15. Previously, we have reported that both Kir2.1 and Kv1.3 are expressed in HMDMs and foam cells 17, 19, 23. Blocking of Kir2.1 (with BaCl_2_) and Kv1.3 (with margatoxin or diclofenac) significantly reduced cholesterol ester (CE) content in macrophage and inhibited the formation of foam cell from macrophage 16, 19. However, the role of Kir2.1 in HMDMs maturation and foam cell formation remains unclear.

Therefore, in this study, we investigated the dynamic expression of Kir2.1 in association with its function in both primary cultured human monocytes and THP‐1–derived macrophages towards the ox‐LDL induced differentiation and foam cells formation, to explore its role in AS formation.

## Materials and methods

### Ethics statement

The use of platelet‐free blood in this study, obtained from healthy volunteers and supplied by the Xi'an Blood Center periodically from March 2011 to April 2013, conformed to the Declaration of Helsinki. The study protocol was pre‐approved by the Human Ethics Committee of the First Affiliated Hospital of Xi'an Jiaotong University, China.

### Cell culture

THP‐1 cells (ATCC, Rockefeller, MD, USA) were maintained at 5 × 10^5^ cells/ml in RPMI‐1640 medium (Gibco, Carlsbad, CA, USA) supplemented with 10% foetal bovine serum (FBS) and 2 mmol/l L‐glutamine, seeded into 24‐well plates, and then differentiated by stimulating with 100 μg/l phorbol 12‐myristate 13‐acetate (PMA; Sigma‐Aldrich, St Louis, MO, USA) for 72 hrs. The THP‐1–derived macrophages were randomly divided into control (without any special treatment) or ox‐LDL [treated with 30 mg/l ox‐LDL with a malondialdehyde content of 39 mmol/l (Chinese Academy of Medical Science and Peking Union Medical College, Beijing, China) for 72 hrs] groups.

Forty‐two platelet‐free blood samples obtained from healthy volunteers (aged between 20 and 40 years) were supplied by the Xi'an Blood Centre periodically from March 2011 to April 2013. Differentiation of cultured human monocytes was established as previously described 17, 24. Briefly, mononuclear cells were separated from the platelet‐free blood by cell density gradient centrifugation, and then cultured in RPMI‐1640 medium containing 10% FBS at 37°C in 5% CO_2_.

The cultured monocytes were randomly divided into three groups: (*i*) Control 5 day Group (C 5d): monocytes were cultured for 5 days without any special treatment; (*ii*) Control 7.5 day Group (C 7.5d): monocytes were cultured for 7.5 days without any special treatment; (*iii*) ox‐LDL treatment group (ox‐LDL): monocytes were cultured for 5 days, then incubated with 30 mg/l (final concentration) ox‐LDL for 60 hrs. Among them, the monocytes separated from a total 15 subjects were sampled for the molecular biology experiment individually, and each group was tested five times. In all experiments, trypan blue assay verified that cell viability was >95%.

### Oil red O staining

Cell smears were prepared by a sequence of 10 min. fixation with 4% paraformaldehyde, rinsing with 60% isopropyl alcohol, 10 min. staining with Oil red O (Sigma‐Aldrich), and then rinsing with 60% isopropyl alcohol and deionized water. Prior to sealing with neutral gum, the slides were stained with haematoxylin–eosin. All steps were performed at room temperature.

### Determination of intracellular lipid content

Cells were collected in 0.5 ml isopropyl alcohol and disrupted with an ultrasonic disrupter (Shanghai Ultrasonic Instrument Factory, Shanghai, China) for 3 min. with a pulse time of 30 sec. and standing time of 30 min. at 4°C. Following centrifugation at 800 × g for 5 min., the supernatant was divided equally and the total cholesterol (TC) and free cholesterol (FC) content was determined using a cholesterol kit (Cayman Chemical Company, Ann Arbor, MI, USA); reactions were measured using a microplate reader (MQX‐200R; Bio‐Tek Instruments, Inc., Winocski, VT, USA) at the excitation wavelength of 565 nm and emission wavelength of 590 nm. The total protein content was determined using a BCA Protein Assay Kit (Shaanxi Pioneer Biotechnology, Xi'an, China). Cholesterol ester was defined as the difference between TC and FC (TC − FC) and was expressed in units of mg/g cellular protein.

### Kir2.1 knockdown with small interfering RNA

The small interfering RNA (siRNA) used to target human Kir2.1 (National Center for Biotechnology Information: NM_000891.2) was synthesized by Life Technologies (Carlsbad, CA, USA). Its sequence was 5′‐TGCTGAAAGAGCACAGGCTCATAGCGGTTTTGGCCACTGACTGACCGCTATGACTGTGCTCTTT‐3′; 5′‐tgctgAAATGTACTGCGCGTGGAGACGTTTTGGCCACTGACTGACGTCTCCACGCAGTACATTT‐3′ was used as scramble siRNA. The Kir2.1 siRNA was subcloned into pcDNA6.2^™^‐GW/EmGFP‐miR plasmids. THP‐1 cells were seeded in 6‐well plates at 5 × 10^5^ cells/well to 70–80% confluence. Lipofectamine^®^ 2000 (Life Technologies) was used according to the manufacturer's instructions to transfect the siRNAs. The gene silencing effect was analysed by RT‐PCR following 48‐hrs transfection.

### RNA extraction and real‐time RT‐PCR

Total cellular RNA was extracted using an RNA _fast200_ kit (Shanghai Flytech Biotechnology, Shanghai, China) according to the manufacturer's instructions, and complementary DNA (cDNA) was synthesized using a RevertAid^™^ First Strand cDNA Synthesis Kit (Fermentas, Foster City, CA, USA). PCR was carried out using Real‐Time PCR Master Mix (Fermentas), with a total 40 cycles of 50°C for 2 min., 95°C for 10 min., 95°C for 15 sec., 60°C for 30 sec. and 72°C for 30 sec. The melting curve was analysed after the amplification, and from 55°C to 95°C, the values were read once every 0.5°C increment. All PCR products were confirmed by agarose gel electrophoresis (30 g/l) with the corresponding specific bands. Primer sequences and fragments of amplified products are listed in Table 1. The copy numbers of the target genes were calculated using the comparative threshold cycle [2^−ΔΔc(t)^] method.

**Table 1:** Primer sequences used for PCR amplification

Gene	Primer sequence (forward, reverse)	Product (bp)
*KCNJ2* (NM_000891.2)	5′‐TCAGAAGAAGACGGTATGAAGTTGG‐3′ 5′‐CAGGCAGAAGATAACCAGCATCC‐3′	231
*ACTB* (NM_001101.3)	5′‐ATCGTGCGTGACATTAAGGAGAAG‐3′ 5′‐AGGAAGGAAGGCTGGAAGAGTG‐3′	179

### Protein extraction and Western Blotting

Cells were lysed with 500 μl ice‐cold macrophage lysis buffer (containing 10 g/l Nonidet P‐40, 100 ml/l glycerol, 50 mmol/l 4‐(2‐Hydroxyethyl)‐1‐piperazineethanesulfonic acid (HEPES), 150 mmol/l NaCl, 1 mg/l aprotinin, 1 mg/l leupeptin, 86 mg/l iodoacetamide and 1 mmol/l phenylmethylsulfonyl fluoride, pH 7.5). The supernatant was collected after centrifugation at 12,000 × g for 10 min. at 4°C. After determining the protein concentration, the samples were mixed with loading buffer and boiled for 5 min. prior to electrophoresis at 4°C on 100 g/l sodium dodecyl sulphate–polyacrylamide gels (100 V, 1.5 hrs), and the 30 V wet method was used for membrane transfer overnight. After blocking with 50 g/l skim milk in Tris‐buffered saline (TBS) at room temperature for 90 min., the membrane was incubated accordingly with rabbit anti‐human Kir2.1 polyclonal antibodies (Alomone Labs, Jerusalem, Israel), rabbit anti‐human SR‐A monoclonal antibodies (Abcam, London, UK), rabbit anti‐human SR‐BI monoclonal antibodies (Abcam), or rabbit anti‐human CD36 monoclonal antibodies (Santa Cruz Biotechnology, Inc., Santa Cruz, CA, USA) overnight at 4°C, and then with 1:500 stabilized goat anti‐rabbit horseradish peroxidase‐conjugated antibody (Pierce, Rockford, IL, USA) at room temperature for 1 hr following TBS and Tween 20 washing for 15 min. four times. An enhanced chemiluminescence detection kit (Pierce) was used to detect these proteins, and protein expression levels were expressed as the ratio of the integrated optical density of Kir2.1 protein to that of β‐actin (ACTB).

### Patch clamp technique

Cells were superfused with bath solution with the following composition (in mmol/l): NaCl 140, KCl 4, CaCl_2_ 2, MgCl_2_ 1, glucose 5, HEPES 10; the pH was adjusted to 7.4 with NaOH. For recording the K^+^ current, a pipette was filled with the following (in mmol/l): KCl 20, K‐Aspirate (to suppress Cl^−^ currents) 115, MgCl_2_ 1, Na_2_ATP 2, ethylene glycol bis (β‐aminoethyl ether)‐*N*,*N*,*N*′,*N*′‐tetraacetic acid (EGTA) 5, HEPES 10; the pH was adjusted to 7.2 with KOH. The recordings were obtained under visual control using a microscope (Olympus, Tokyo, Japan). Operation of the pipette was controlled by electrical micromanipulators (Sutter Instruments, Novato, CA, USA). An Axopatch 700B amplifier (Axon Instruments, Foster City, CA, USA) and Digidata 1320 digital–analog converter (Axon Instruments) were used to record the electrophysiological signal. Offset potentials were nulled directly before the formation of a seal. No leak subtraction was made. Cell capacitance (in pF) was made from whole‐cell capacitance compensation. The effective corner frequency of the low‐pass filter was 0.5–5 kHz. The frequency of digitization was at least twice that of the filter. Data were stored and analysed with commercial pCLAMP9.0 software (Axon Instruments). All experiments were performed at room temperature. The K^+^ current was eluted with a stepwise protocol.

### Statistical methods

Data are presented as the mean ± SE. The homogeneity of variance was tested prior to comparisons among three groups using one‐way anova. The Student–Newman–Keuls test was conducted between any two groups after anova. The significance level of all hypothesis testing was set as α = 0.05. SPSS 13.0 statistical software (SPSS Inc., Chicago, IL, USA) was used for data analyses.

## Results

### Morphology of THP‐1–derived macrophages and foam cells

**Figure 1:**
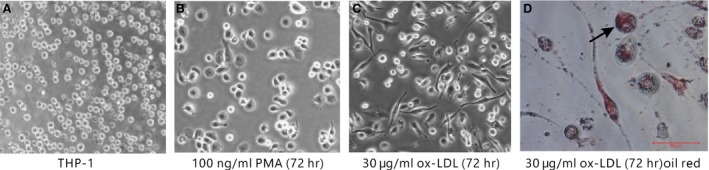
Differentiation of THP‐1 cells. THP‐1–derived macrophages (**A**) following 72‐hrs incubation with (**B**) 100 μg/l PMA or (**C**) 30 mg/l ox‐LDL (×10). (**D**) Oil Red O‐stained foam cells, numerous lipid granules presented in cytoplasm (black arrows; ×40).

THP‐1 cells showed the potential to differentiate into macrophages and foam cells. In the presence of PMA, most THP‐1 cells attached to the bottom of the Petri dish and differentiated into macrophages (Fig. 1A and B). Following ox‐LDL treatment, the THP‐1–derived macrophages took up lipids and differentiated into foam cells (Fig. 1C and D).

### Kir2.1 expression in THP‐1–derived macrophages and foam cells

**Figure 2:**
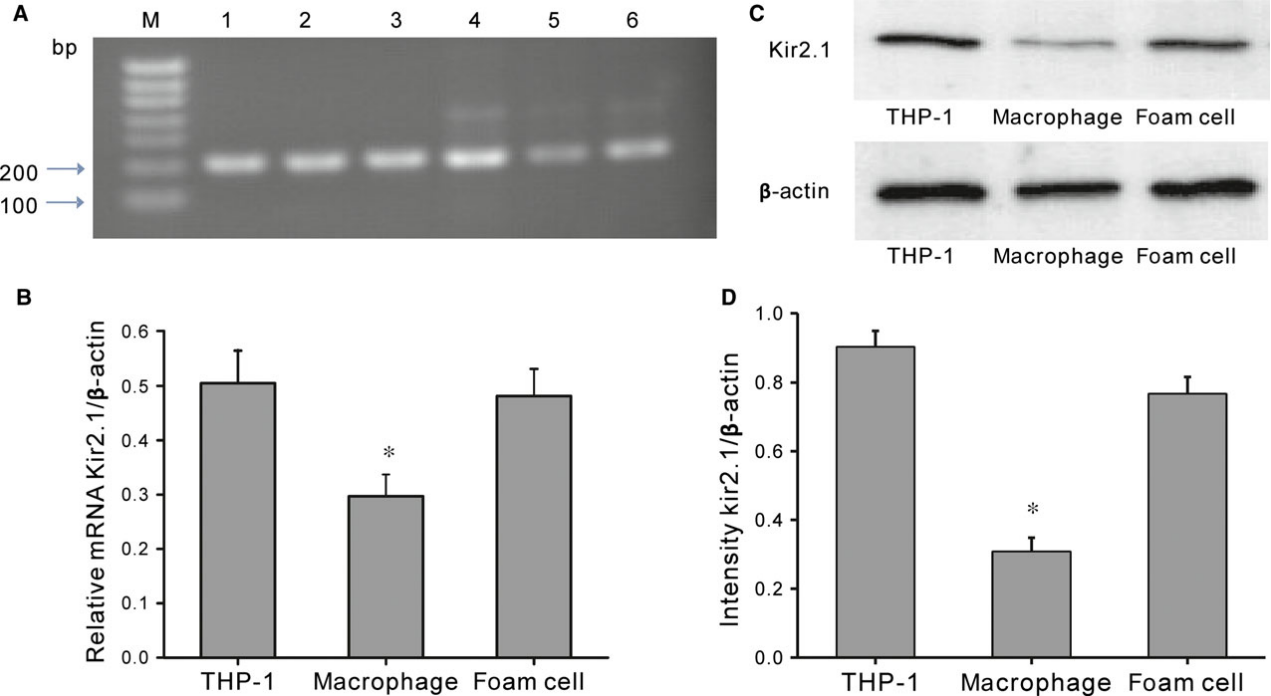
Kir2.1 expression in THP‐1 and differentiated cells. (**A**) Agarose gel of RT‐PCR products. M, 100‐bp DNA ladder; Lane 1–3, GAPDH (205 bp); Lane 4–6, Kir2.1 (199 bp). (**B**) *KCNJ2* mRNA expression. *P < 0.05 *versus* THP‐1 or foam cells. (**C**) Western blotting of Kir2.1 protein expression. (**D**) Kir2.1 protein expression. *P < 0.05 *versus* THP‐1 or foam cells. Values are the mean ± SE.

**Figure 3:**
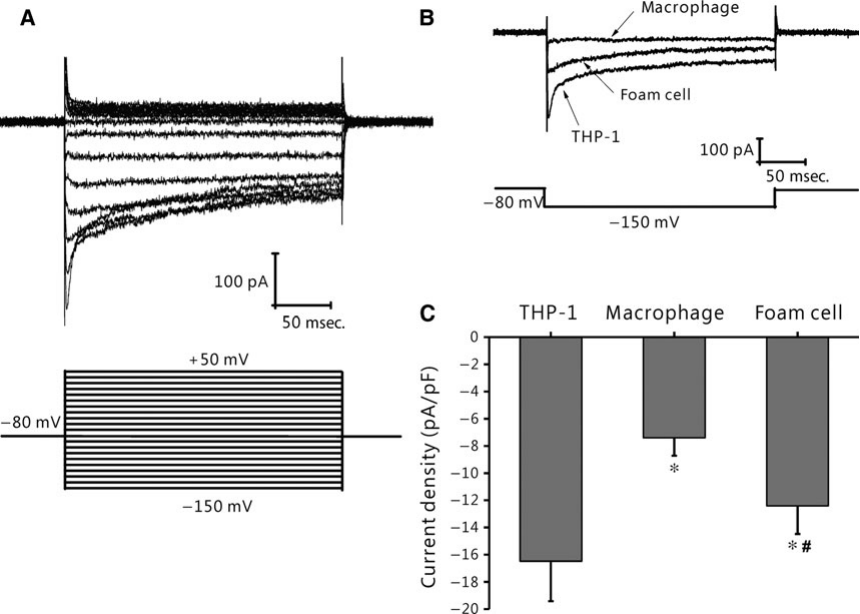
Development change in Kir2.1 currents in THP‐1 cells. (**A**) Typical traces of Kir2.1 currents recorded from THP‐1 cells. Membrane potential was held at −80 mV and pulse potentials were applied from −150 mV to +50 mV, stepped 10 mV as indicated. (**B**) Representative traces of Kir2.1 currents from THP‐1 cells, THP‐1–derived macrophages, and foam cells. (**C**) Comparison of current amplitude at −150 mV of Kir2.1 currents in THP‐1 cells (*n* = 13), macrophages (*n* = 6), and foam cells (*n* = 8). *P < 0.001 *versus* THP‐1 cells; *,#*P* < 0.05 *versus* THP‐1 cells or macrophages. Values are the mean ± SE.

The dynamic expression level of Kir2.1 was detected during THP‐1 cell differentiation (Fig. 2A–D). RT‐PCR showed that the expression of *KCNJ2* mRNA was significantly decreased (41.2%, *P* < 0.05, *n* = 5) at 72 hrs after PMA treatment, and was increased by 1.6 times in foam cells compared to that in the macrophages (*P* < 0.05). Consistently, Kir2.1 protein levels were decreased significantly in macrophages after PMA stimulation (44.2% decrease, *P* < 0.05, *n* = 5; Fig. 3C), whereas that in the foam cells was 1.6 times higher than that in the macrophages (*P* < 0.05; Fig. 3C).

### Changes of Kir2.1 inward rectifier current in THP‐1–derived macrophages and foam cells

THP‐1 cells express Kir2.1 currents (Fig. 3A). As shown in Figure 3B, Kir2.1 currents were largely decreased after cells were treated with PMA, whereas they were increased upon THP‐1 cells differentiating into foam cells. The peak Kir2.1 current densities of the THP‐1 cells, foam cells and macrophages measured at −150 mV were −16.8 ± 2.93 pA/pF (*n* = 13), −12.41 ± 2.07 pA/pF (*P* < 0.001 *versus* THP‐1, *n* = 6) and −7.39 ± 1.32 pA/pF (both *P* < 0.05 *versus* THP‐1 cells or macrophages, *n* = 8) respectively (Fig. 3C).

### Kir2.1 knockdown in THP‐1 cells inhibited foam cell formation

**Figure 4:**
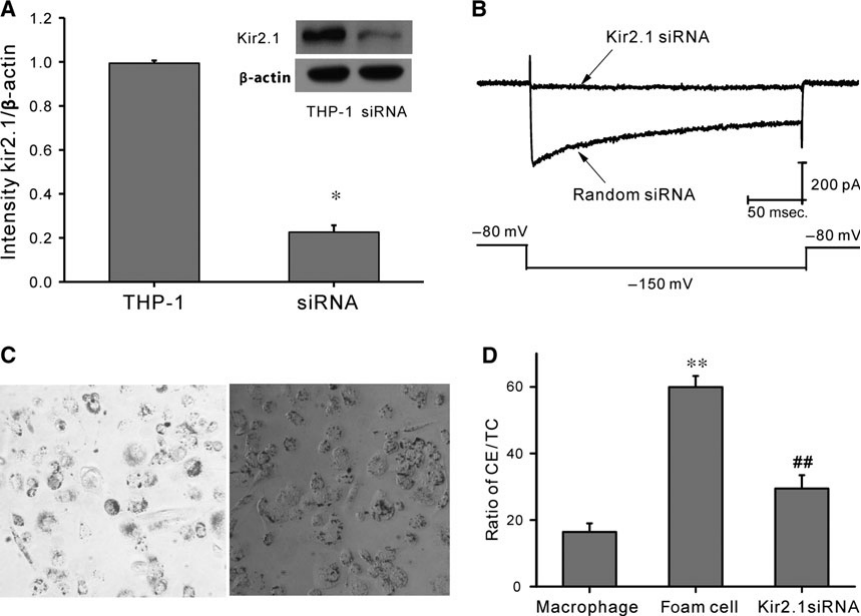
Kir2.1 silencing using Kir2.1 siRNA. (**A**) Typical protein expression gels (top) and histogram (bottom) showing Kir2.1 protein expression following transfection of Kir2.1 and scrambled siRNAs. *P < 0.05, scrambled siRNA *versus* Kir2.1 siRNA. (**B**) Representative traces of Kir2.1 currents from THP‐1 cells treated with Kir2.1 siRNA (top) or scrambled siRNA (bottom). (**C**) Oil red O staining of THP‐1–derived macrophages treated with 30 mg/l ox‐LDL (×100). Left: Control group; right: Kir2.1 siRNA group. (**D**) Histogram showing the ratio of intracellular CE in macrophages, foam cells, and Kir2.1 siRNA‐transfected macrophages. **P < 0.01 *versus* macrophages; ##*P* < 0.01 *versus* foam cells. Values are the mean ± SE.

Given their proliferative features, THP‐1 cells were used as a model to demonstrate the effects on Kir2.1 inhibition in macrophage differentiation. Kir2.1 protein in THP‐1 cells was knocked down with specific siRNA. After 48‐hr siRNA transfection, the expression of Kir2.1 protein was significantly down‐regulated (being only 22.7% of that in the control, *P* < 0.05; Fig. 4A). Consistently, Kir2.1 current density was decreased significantly after cells had been transfected with the specific siRNA, but was not affected by the scrambled siRNA (Fig. 4B). The results suggest that Kir2.1 in THP‐1 cells was successfully and efficiently silenced by the siRNA.

In the Kir2.1 knockdown THP‐1 cell model, although the cells still could be transformed into macrophages in response to PMA treatment, the cellular lipid uptake capability was lost after the differentiation. Following ox‐LDL treatment, the Kir2.1 knockdown macrophages contained much less lipids than the control foam cells (Fig. 4C). The CE/TC ratio (Fig. 4D) was significantly decreased to 29.46 ± 2.01% compared with that of the control (59.94 ± 2.56%, *P* < 0.05). Therefore, Kir2.1 knockdown inhibits the formation of THP‐1–derived macrophages into foam cells.

### Role of Kir2.1 in human macrophage maturation and foam cell formation

**Figure 5:**
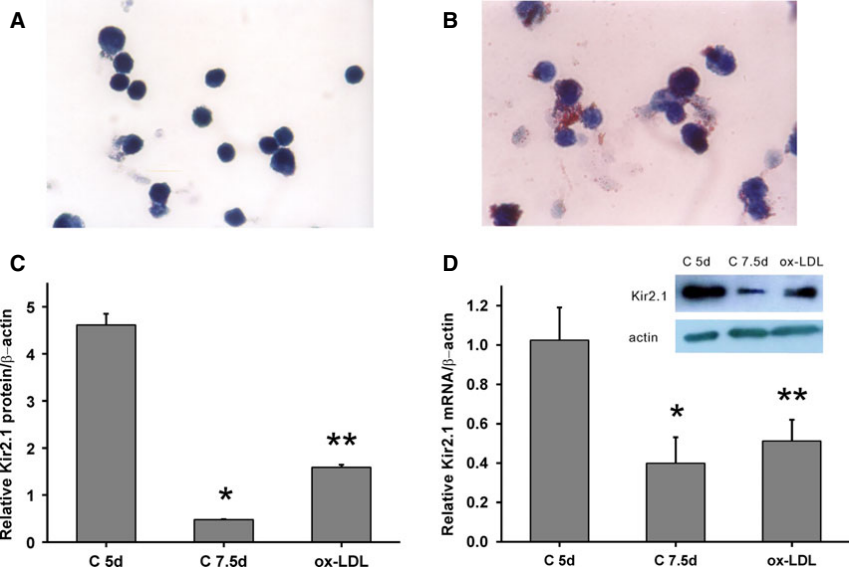
Kir2.1 expression in human monocyte‐derived macrophages. Cultured human monocyte‐derived macrophages (**A**) and foam cells (**B**) stained with Oil red O (×100). (**C**) *KCNJ2* mRNA expression. *, **P < 0.05 *versus* the C 5d group; **P > 0.05 *versus* the C 7.5d group. (**D**) Typical protein expression gels (top) and histogram (bottom) showing the expression of Kir2.1 protein. *, **P < 0.001 *versus* the C 5d group; **P = 0.001 *versus* the C 7.5d group. Values are the mean ± SE.

A human primary macrophage differentiation model relevant to AS in humans was used as previously described 16. After 5‐day culture, human monocytes were attached to the bottom of the Petri dish, and their shape changed from round to irregular, indicating the occurrence of macrophage transformation (Fig. 5A) 25, 26. After further treatment with ox‐LDL for 60 hrs, the macrophages were transformed into foam cells, which were observed under an optical microscope through the presence of red lipid granules following Oil Red O staining (Fig. 5B).

Kir2.1 expression was investigated at both mRNA and protein levels. During macrophage differentiation, *KCNJ2* mRNA expression levels were significantly decreased by >60% compared to that of cells in the C 5d group (*P* < 0.05; Fig. 5C). However, following ox‐LDL treatment, *KCNJ2* mRNA expression was only slightly increased (by approximately 1.3 times compared with the C 7.5d group, *P* > 0.05, Fig. 5C). Concurrently, Kir2.1 protein levels were also decreased during macrophage differentiation (by 90% compared with cells in the C 7.5d group, *P* < 0.001). Whereas Kir2.1 protein levels were 3.3 times higher in the ox‐LDL group than in the C 7.5d group (*P* = 0.001, Fig. 5D).

**Figure 6:**
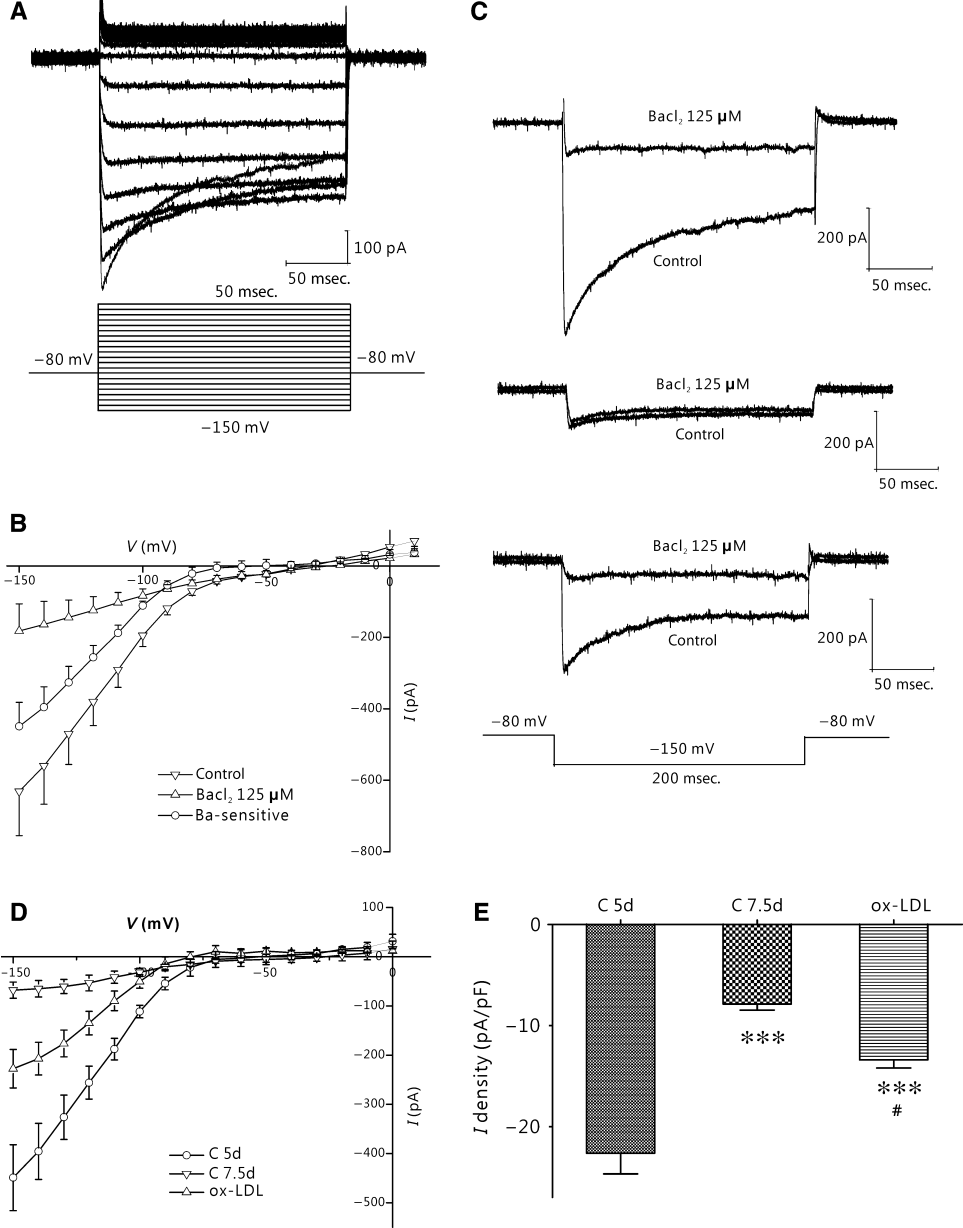
Development change Kir2.1 currents in human monocyte‐derived macrophages. (**A**) Typical traces of Kir2.1 currents recorded from macrophages in the C 5d group. Membrane potential was held at −80 mV and pulse potentials were applied from −150 mV to +50 mV, stepped 10 mV as indicated. (**B**) I‐V curves showing that Kir2.1 currents in C 5d group macrophages were inhibited by BaCl_2_. The BaCl_2_‐sensitive current was calculated by subtracting the current in the presence of 125 μmol/l BaCl_2_ from the control current (*n* = 5 per group). (**C**) Representative traces of Kir2.1 currents pre‐ and post‐BaCl_2_ application from macrophages in the C 5d (top), C 7.5d (middle) and ox‐LDL groups (bottom). (**D**) Relative I‐V curves of BaCl_2_‐sensitive current in the C 5d, C 7.5d and ox‐LDL groups (*n* = 5 per group). (**E**) Comparison of the current amplitude at −150 mV of Kir2.1 currents in the C 5d (*n* = 47), C 7.5d (*n* = 28) and ox‐LDL (*n* = 18) groups. ***P < 0.001 *versus* the C 5d group; #*P* < 0.05 *versus* the C 7.5d group. Values are the mean ± SE.

The whole‐cell Kir2.1 current in the primary monocyte‐derived macrophages was compared among the C 5d, C 7.5d and ox‐LDL groups using the patch clamp technique. The inward rectifying K^+^ currents were incited by stepwise 200 msec. depolarizing pulses from −150 mV to +50 mV with a 10‐mV step. Figure 6A illustrates a typical inward rectifying K^+^ current of macrophages (C 5d). BaCl_2_ (125 μmol/l), a selective Kir2.1 blocker, inhibited most of the inward rectifying current component (Fig. 6B). The BaCl_2_‐sensitive Kir2.1 current was calculated by subtracting the current in the presence of BaCl_2_ from the total inward rectifying K^+^ current.

To identify the role of Kir2.1 in the different stages of macrophage differentiation, we compared the BaCl_2_‐sensitive Kir2.1 currents in the C 5d, C 7.5d and ox‐LDL groups. Figure 6C depicts a representative inward rectifier K^+^ current and blockage with 125 μmol/l BaCl_2_ in the groups. A larger proportion of BaCl_2_‐sensitive current was detected in the C 5d and ox‐LDL groups, but not in the C 7.5d group. Figure 6D shows the composite data, Figure 6E the current–voltage (I‐V) curve and peak current density of the BaCl_2_‐sensitive current in the three groups. The peak Kir2.1 current density, measured at −150 mV, was decreased significantly in the C 7.5d group (−7.88 ± 0.60 pA/pF, *n* = 28, *P* < 0.001) compared to that in the C 5d group (−22.61 ± 2.1 pA/pF, *n* = 47; Fig. 6F). However, the current density remained partially in the ox‐LDL group (−13.39 ± 0.80 pA/pF, *n* = 18, *P* < 0.001 *versus* C 5d, *P* < 0.05 *versus* C 7.5d respectively, Fig. 6F).

### Kir2.1 knockdown regulates the expression of scavenger receptors in THP‐1–derived macrophages

**Figure 7:**
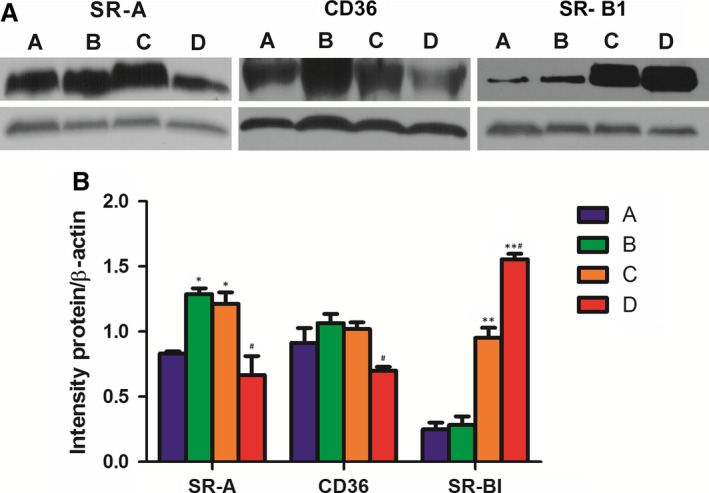
Change scavenger receptors in THP‐1–derived macrophages. (**A**) Western blotting of SR‐A, CD36 and SR‐BI protein expression. (**B**) Relative expressions of SR‐A, CD36 and SR‐BI proteins. (a) Macrophages; (b) foam cells; (c) Kir2.1 siRNA‐transfected macrophages; (d) Kir2.1 siRNA‐transfected macrophages treated with 30 mg/l ox‐LDL. *P < 0.05 and **P < 0.001 *versus* the macrophages; #*P* < 0.05 *versus* the Kir2.1 siRNA‐transfected macrophages. Values are the mean ± SE.

The dynamic change in the scavenger receptors of SR‐A, CD36 and SR‐BI in THP‐1–derived macrophages was detected under different conditions. After incubating with 30 mg/l ox‐LDL for 72 hrs, protein expression of SR‐A of the THP‐1–derived macrophages was significantly increased by over 1.5 times, compared to that of macrophages (*P* < 0.05), whereas that of CD36 and SR‐BI were only slightly but not significantly increased (both by approximately 1.1 times compared with the macrophages, *P* > 0.05, Fig. 7).

After knockdown of Kir2.1 with siRNA, the protein levels of SR‐A and SR‐BI were all significantly increased (approximately 1.5 times and 3.3 times compared with the macrophages, *P* < 0.05 and *P* < 0.001, respectively, Fig. 7), but that of CD36 had an insignificant change (increased by 1.1 times, *P* > 0.05, Fig. 7). However, following ox‐LDL treatment with the Kir2.1 knockdown macrophages, the SR‐BI expression was further increased by over 6.2 times, compared to that in macrophages (*P* < 0.001, Fig. 7) and over 1.6 times compared to that in the Kir2.1 knockdown macrophages (*P* < 0.001, Fig. 7). Contrarily, both SR‐A and CD36 protein levels were significantly reduced, respectively, by 45.1% and 31.3% compared with that in the Kir2.1 knockdown macrophages (*P* < 0.05) and even slightly lower than that in macrophages (*P* > 0.05, Fig. 7). These results suggest that Kir2.1 silencing inhibited the foam cell formation probably by significantly up‐regulating the SR‐BI expression together with interfering the interactions of SR‐A and CD36 with ox‐LDL in the THP‐1–derived macrophages.

## Discussion

### Main findings

In this study, we investigated the key role of Kir2.1 in macrophage lipid uptake and in the formation of foam cells, and demonstrated that (*i*) Kir2.1 is down‐regulated and loses its functionality in mature macrophages derived from either THP‐1 cells or human primary monocytes, but remains active under ox‐LDL stimulation and a significant up‐regulation of SR‐A in THP‐1–derived foam cells; (*ii*) Kir2.1 knockdown in THP‐1–derived macrophages prevents lipid uptake and foam cell formation. (*iii*) Kir2.1 knockdown up‐regulates the SR‐BI expression significantly and interferes with both interactions of SR‐A and CD36 to ox‐LDL in the THP‐1–derived foam cells. The current results may reveal the critical role of Kir2.1 in the lipid uptake is modulated by the expression of scavenger receptors in macrophages.

### Monocyte‐derived macrophage and foam cell model

THP‐1 is a human monocytic leukaemia cell line that closely resembles the native monocyte‐derived macrophages in many respects, such as surface receptors and ionic conductance 27, 28, 29. THP‐1 cells have several ion currents, such as the delayed rectifier K^+^ current, inward rectifying K^+^ current, and Ca‐activated K^+^ current 30. Although there are some limitations, THP‐1 remains a well‐characterized, easily accessible model for studying macrophage activation or differentiation. Here, we showed that THP‐1 cells express BaCl_2_‐sensitive, inward rectifying Kir2.1 conductance. The Kir2.1 currents were reduced after cells had been cultured for 60 hrs. However, the Kir2.1 currents were greatly increased following ox‐LDL treatment. Therefore, Kir2.1 may be critical for maintaining the lipid uptake and foam cell formation of THP‐1–derived macrophages.

To understand the role of macrophages in the lipid uptake and foam cell formation in humans, fresh separated primary human monocytes from healthy donor blood were used in this study. We showed that KCNJ2 mRNA and protein are expressed in human primary monocyte‐derived macrophages, and the typical inward rectifier currents were sensitive to BaCl_2_, a selective Kir2.1 blocker. Combined with the mRNA and protein expression results, we may confirm that Kir2.1 is expressed in human primary monocyte‐derived macrophages. We also observed that C 5d macrophages expressed maximal Kir2.1 currents, which were diminished after the cells had been cultured for 60 hrs. Compared to the untreated macrophages, the Kir2.1 currents were much higher in the ox‐LDL–treated cells, suggesting that the Kir2.1 current is activated in association with cellular lipid uptake function and foam cell formation.

### Kir2.1 augments ox‐LDL uptake in macrophages

Our previous results indicate that Ba^2+^ can block Kir2.1, resulting in decreased TC and CE in ox‐LDL–stimulated macrophages and inhibited foam cell formation 16. It has been suggested that Kir2.1 may be involved in the lipid uptake of macrophages. However, Kir2.1 blockage with Ba^2+^ is not exclusive 31, 32, 33. Therefore, we silenced Kir2.1 expression using specific siRNA to further investigate the role of Kir2.1 in cellular ox‐LDL uptake and foam cell formation. We found that silencing Kir2.1 had no effect on the transformation of THP‐1 cells to macrophages under PMA stimulation, but attenuated ox‐LDL uptake and foam cell formation. Thus, this study indicted firstly that Kir2.1 may mediate ox‐LDL uptake and foam cell formation from macrophages.

### Possible underlying mechanism

Kir2.1 facilitates K^+^ movement into, rather than out of, the cell to maintain a balanced membrane potential. When the Kv1.3 current sets the membrane potential at −50 to −60 mV, the Kir2.1 current shifts the potential to more negative values, closer to the K^+^ equilibrium potential. During human macrophage‐derived foam cells formation, the newly expressed Kir2.1 may generate a window Ca^2+^ current (mediated by alpha 1H T‐type Ca^2+^ channels) *via* their hyperpolarizing effect on the membrane potential and cause intracellular Ca^2+^ to increase 34, which can trigger macrophage differentiation into foam cells *via* activation of the calcineurin pathway and the expression of specific proteins 22, 35. Therefore, we speculate that blocking Kir2.1 could depolarize the resting membrane potential 19, decrease Ca^2+^ influx, and consequently inhibit foam cell formation 16, 19.

In this study, we observed that the current density augmentation alteration may be related to increases of expressions of Kir2.1 and SR‐A during foam cell formation (both *P* < 0.05), which can promote macrophages to uptake ox‐LDL effectively 11, 36 and differentiate into foam cells. Although Kir2.1 silencing increased all scavenger receptor expressions, including SR‐A (*P* < 0.05), CD36 (*P* > 0.05) and SR‐BI (*P* < 0.001), ox‐LDL could revised their expressions. In the Kir2.1 knockdown macrophages, the SR‐BI expression was further increased (by over 6.2 times compared to that of macrophages, *P* < 0.001), but both the SR‐A and CD36 expressions were significantly decreased (by 45.1% and 31.3% compared with the Kir2.1 knockdown macrophages, respectively, *P* < 0.05), and even slightly lower than that of macrophage (*P* > 0.05). Thus, decrease in ox‐LDL intake 36 together with increase in cholesterol out‐flow 10, 37, eventually inhibited macrophage differentiating into foam cell. These data might explain why the Kir2.1 silenced THP‐1 derived macrophages partially lost its ability of lipid uptake. Although the exact signalling pathway remains unknown, we speculate that Kir2.1 plays a crucial role in human macrophage‐derived foam cells formation by modulating the expression and function of the scavenger receptors.

## Conclusions

This study demonstrated the roles of Kir2.1 in human macrophage differentiation, lipid uptake and foam cell formation. During its differentiation, the macrophage with up‐regulated Kir2.1 and SR‐A remains hyperactive in lipid uptake and foam cell formation, which could be attenuated by Kir2.1 silencing together with a significant SR‐BI up‐regulation.

## Conflicts of interest

We declare that we have no conflict of interest.
